# Supplementary material for: Electrochemical Disinfection of Dental Implants Experimentally Contaminated with Microorganisms as a Model for Periimplantitis
Source: J Clin Med. 2020 Feb 9;9(2):475. doi: 10.3390/jcm9020475 (PMC7074531; doi:10.3390/jcm9020475)
Supplement: Supplementary file 1 [file jcm-09-00475-s001.pdf]

Table S1. Overview of disinfection experiments. Sample size and groups are given for organisms and treatment methods applied (n.d., not determined). Embedment methods used: silicone, polyurethane foam and bovine ribs.

Microbial species	Curette	Air abrasion	BDD treatment
**Bacteria**			
B. pumilus	n.d.	n.d.	3 independent biological replicates for 3 embedment methods and 4 time points[a)], respectively
B. subtilis	n.d.	n.d.	3 independent biological replicates for 3 embedment methods and 4 time points[a)], respectively
E. faecalis	3 independent biological replicates for 3 embedment methods	3 independent biological replicates for 3 embedment methods	3 independent biological replicates for 3 embedment methods and 4 time points[b)], respectively
R. mucosa	n.d.	n.d.	3 independent biological replicates for 3 embedment methods and 4 time points[b)], respectively
S. sanguinis	n.d.	n.d.	3 independent biological replicates for 3 embedment methods and 3 time points[b)], respectively
S. epidermidis	n.d.	n.d.	3 independent biological replicates for 3 embedment methods and 5 time points[c)], respectively
**Yeasts**			
C, albicans	n.d.	n.d.	3 independent biological replicates for 3 embedment methods and 5 time points[c)], respectively
C. dubliniensis	3 independent biological replicates for 3 embedment methods	3 independent biological replicates for 3 embedment methods	3 independent biological replicates for 3 embedment methods and 4 time points[c)], respectively
**Mixture of microorganisms**			
Multi-species natural biofilm	3 independent biological replicates for 3 embedment methods	3 independent biological replicates for 3 embedment methods	3 independent biological replicates for 3 embedment methods and 4 time points[b)], respectively

a) 0, 10, 20, 30, 60 min

b) 0, 5, 10, 15 min

c) 0, 5, 10, 15, 20 min
